# Gardens as multispecies public spaces in Georgian modernity

**DOI:** 10.1177/25148486251371535

**Published:** 2025-10-14

**Authors:** Paul Manning

**Affiliations:** Department of Anthropology, Trent University, Peterborough, Canada

**Keywords:** Gardens, multispecies ethnography, infrastructures, Georgia, colonialism

## Abstract

The Kutaisi “Boulevard” (1848) was the first public “park” in Georgia, and quickly became the social and intellectual center of this small, peripheral Georgian city. Although a physically small, and perhaps from an outsider perspective, unremarkable plot of greenspace, as a greenspace, it became a public place central to the imagining of associated literary publics, and finally, as an infrastructure embodying an aspirational claim to modernity, its by turns dusty or muddy garden paths could also be found fault with as being materially a relatively abject incarnation of modernity, something which was felt to always be better incarnated somewhere else. In these critiques of the garden as infrastructure for “liberal walking”, the dusty or muddy paths of the garden were of more interest than the plants. Although all gardens are centres of what Hartigan calls “plant publics”, for these critics, the multispecies publics of the garden (humans and nonhumans) came to be an additional sign of the abjection of infrastructures embodying a failed modernity.

## Central Boulevard

*Kutaisi's Central Boulevard was built in 1848. It was a large garden with straight tree-lined walks with irises, box trees, flowerbeds and tall trees, whose leafy branches made them excellent shade trees. Here too there was a café and a dance ground, where music was played every Sunday. Most of the garden was covered with green ryegrass. There were stone or wood benches for those who came to walk in the Boulevard*.

*It's not clear who was the original planner of the garden, but the type of garden allows us to assume that the gardener was French. It's possible that it was the Frenchman Janio, who designed the flower garden near Khareba Church.*

*This garden was called “Boulevard”, or in a more idiosyncratically Kutaisian way “Gulvard”* [heart-rose], *it was often compared to “Parliament”, the “Athenian Agora”, or the “Roman Forum”, because it soon became the centre of the city's social and cultural life, where many essential issues were discussed. As publicist and public figure Samson Pirtskhalava wrote: “ If you wanted to see someone, that's where you would meet. Here was held buying and selling things, borrowing money, match-making, here debates were held; there was exchange and exhibition for people, beauty, fitness, and clothes. The garden as always full of people, day and night, on the weekends.”*

*In 1948* *on the east side of the garden, they built an entrance with columns. The Central Boulevard still maintains its function as a favourite place for the inhabitants of Kutaisi to gather together. At the edge of the garden *[*baghis k′ide*, referring to a specific sidewalk space of sociability just outside the garden fence] *even today are discussed all issues of concern, beginning with world politics and ending with piquant love stories. *Official plaque on the Kutaisi Central Boulevard

**Figure 1.**
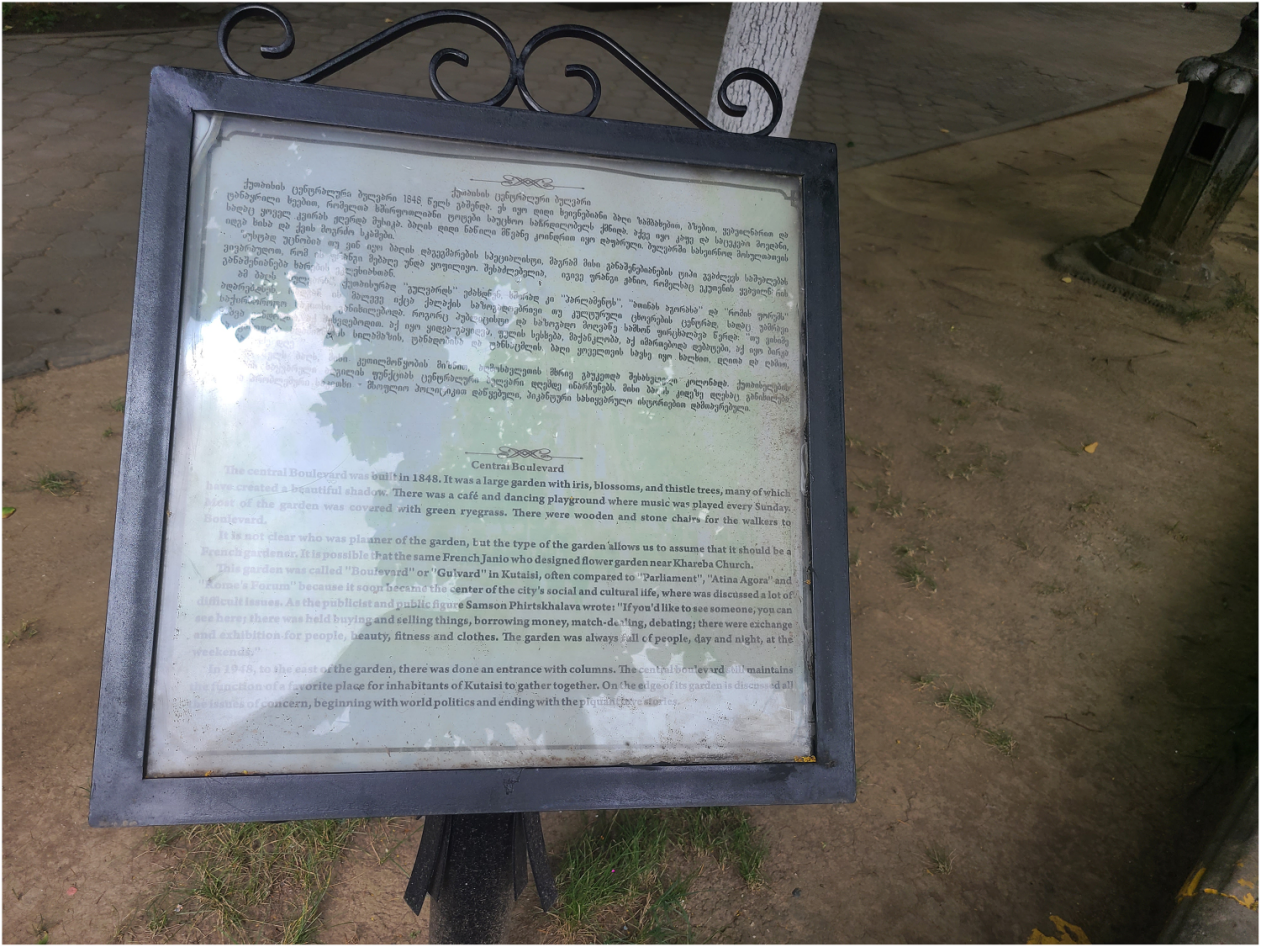
Historical plaque on the Kutaisi Central Boulevard (author photo 2022).

Figure 1 Such is the text on an official plaque on the Kutaisi “Boulevard.” This plaque is one of some 16 plaques that attend the many statues in the garden, making it one of the most densely packed historical spaces in Georgia. Much more history than one would expect from a town around one 10^th^ the size of Georgia's capital, Tbilisi. But, though small, Kutaisi, a student town of the Western province of Imereti, has always been at the forefront of Georgia's intelligentsia movements since the mid nineteenth century, as witnessed by the statues and plaques of all these famous writers, a garden whose walks and benches these same people once frequented, where they held their debates and discussions, according to the myth presented in this plaque.

**Figure 2.**
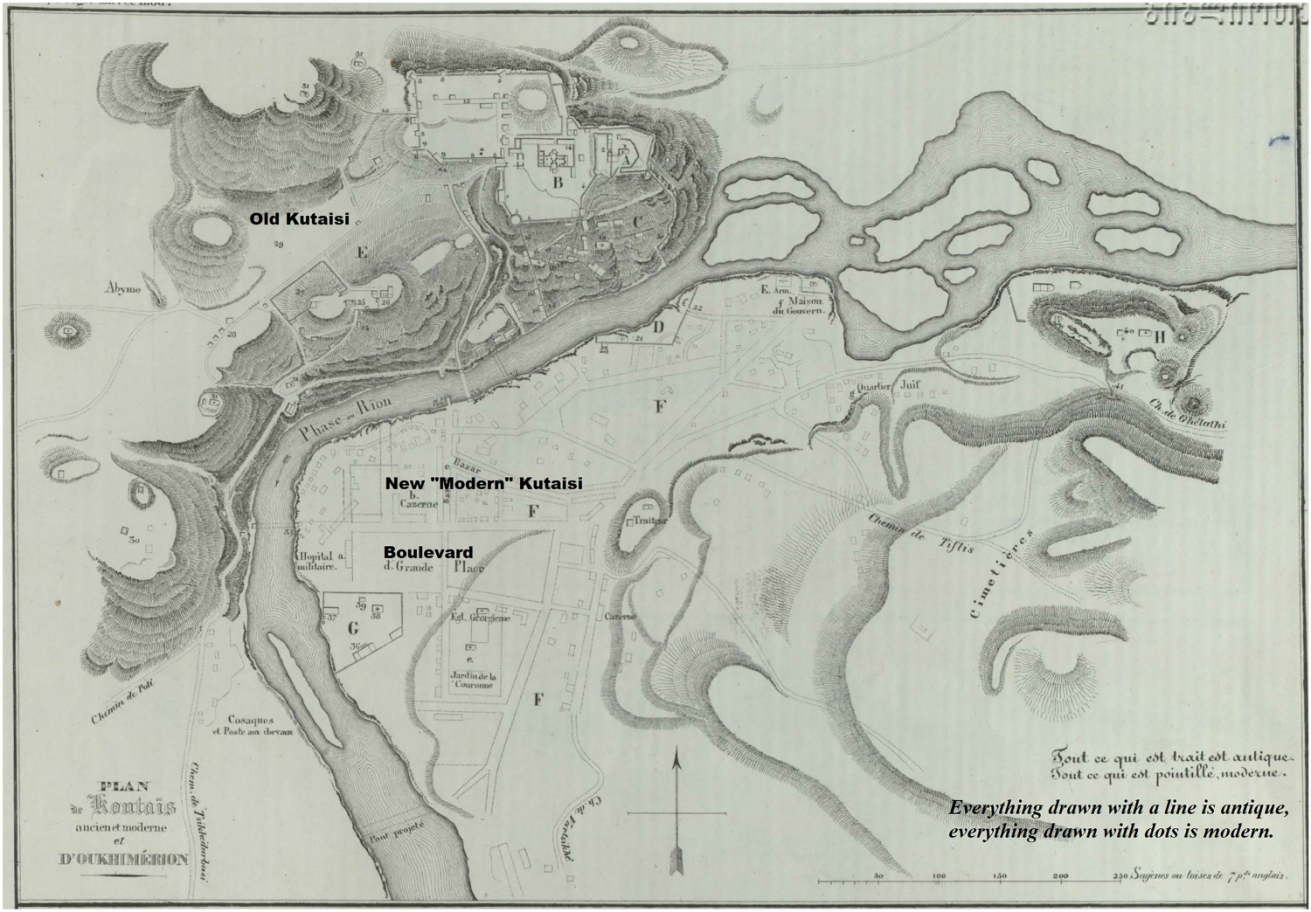
This annotated map of Kutaisi from Dubois de Montpéreux (1843a: no page numbers) labels the space that would later become the Boulevard as “Grande place” [main square] and bears a caption (lower right) that shows the clear demarcation of the city into “ancient” and “modern”: T*out ce qui est trait est antique, tout ce qui est pointille, modern* (“Everything drawn with a line is antique, everything drawn with dots is modern”).

The plaque draws attention to the exceptional status of this central garden, its peculiar name, its mythic centrality to urban public life in all its phases. The so-called “Boulevard” (pronounced variously *bulvari, bulvardi,* also given the Georgian folk etymology *gulvardi* [“heart-rose”]) was officially created in 1848. There is no disagreement that this is the first public garden in Georgia, which became the centre of the new “modern” city under Russian colonial rule (starting 1810–1820 in Kutaisi) Figure 2.

## The Boulevard

*Kutaisi at that time* [sc. in the author's youth, the 1880s] *was somewhere between a city and a village, a city and a garden….If you were to see the city from afar, it would have seemed to you to be one big garden… The social life of Kutaisi was equally picturesque. With strange colours and beautiful forms. In this social life the city garden (the so-called “Boulevard”* [bulvari]*) played a big role. If you wanted to see someone, that's where you would meet…. The garden was always filled with people, by day and night….* Pirtskhalava, 1988: 15)

The introduction to the serialized story “Edisher Kelbakiani” (written by prominent liberal Kutaisi writer Giorgi Tsereteli. (1887)) is perhaps the first literary description of the mythological status the Boulevard had for several generations of West Georgian [Imeretian] Georgian intelligentsia:

Had Kutaisi's Boulevard [*bulvari*] existed in the time of the Romans, now we would without a doubt have a description of it from Sallust or Tacitus. This is Imereti's national council. The Romans would have called it a “Forum”. Nowadays however the Imeretians apparently could not think up a name for it..... [So] they call it a “Boulevard” in a foreign language. … Kutaisi residents consider it “festive”, as they say, to go for a stroll on this Boulevard. A day where you can’t enter the Boulevard and go for a walk there is not a good day. Every place where you step on this Boulevard has its very own specific history. (Tsereteli, 1887: 1)

This Boulevard was a “festive” space for all manner of ways of being in public, strolling and promenading in the latest fashions, seeing and being seen, walking and talking, or sitting on benches and talking, gossiping, and so on (ibid.). For this generation of Georgian intelligentsia Kutaisi's Boulevard becomes a mythic centre, the proper home of *the public* par excellence (Nihil, 1894: 4). For Georgian intelligentsia of the largely liberal generation of the 1860s-1870s, most of whom were, like Giorgi Tsereteli, educated in Kutaisi, the Boulevard was the emblematic centre of liberal models of publicness, and when they wrote feuilletons about public life in Kutaisi, Tbilisi, Telavi, it was about flânerie on boulevards and in gardens that they often wrote about (Manning, 2022, 2023). Georgian flaneurs did not botanize on the asphalt, they did so on garden paths.

**Figure 3.**
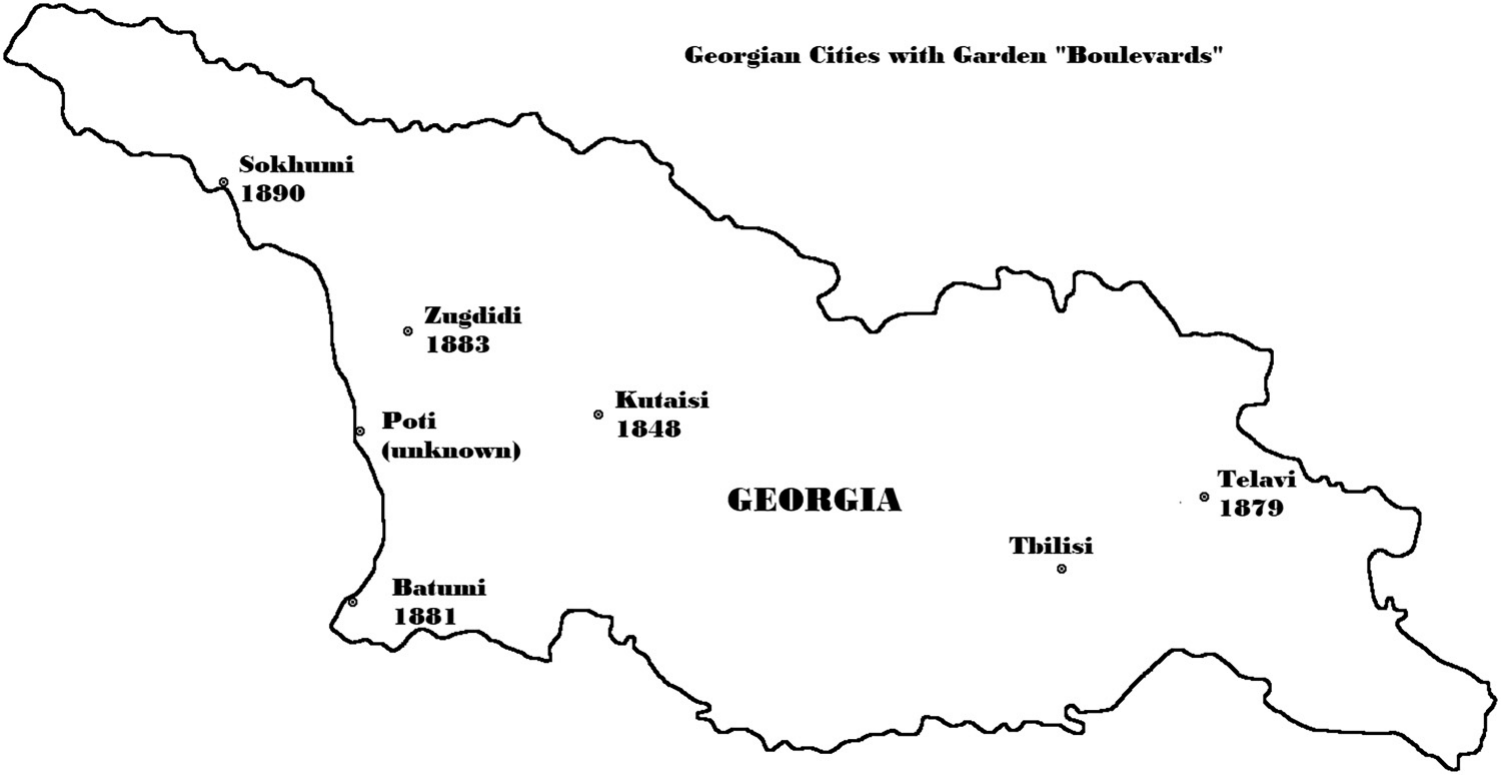
Georgian cities with “boulevards” in the late nineteenth century.

For these liberal Kutaisi intelligentsia, the Kutaisi Boulevard was the founding form of a new kind of central public space, a multispecies assemblage of trees, walks, benches, people, practices like strolling and talking, of a piece with the surrounding streets and their shops and cafes. The boulevard form, beginning with Kutaisi, soon became stabilized enough to become portable to other Georgian cities (Telavi, Batumi, Zugdidi, Sukhumi and Poti). The term boulevard also came to be a shorthand for the intelligentsia or “publics” (the terms were synonymous, Manning, 2012: 89) of that city who gathered and talked on these various boulevards (Sano, 1889), and also, in Tsereteli's serialized story, the proper place to begin any narrative about Kutaisi. Thus the Boulevard, understood as a European (French) garden (created by a mythic anonymous French gardener) became the emblematic centerpiece of any Georgian city's aspirations to “European” style urban modernity and publicness Figure 3.

There were many things one might do spending the day on the Kutaisi Boulevard, strolling, promenading, sitting, talking. Unlike Tbilisi's plebeian gardens of Ortachala (Manning, 2023), drinking wine and feasting was not one of them. Instead, this space was free from the ubiquitous hegemony of Georgian drinking rituals and hospitality. Here instead by 1887 one could enjoy there the first Georgian soft drinks, the fizzy fruit-flavoured, “artificial mineral waters” of Mitrophane Laghidze (Manning, 2012b: 124–127):

Lo, this noteworthy “Boulevard” one evening was as usual filled with strollers. The people gathered like flies in front of yellow painted kiosk. Approximately 100 mouths dried from the heat smacked their lips drinking seltzer water bubbling in glasses and mixed inside sweetened raspberry juice. Sometimes one hand would grab a glass filled from a fizzy tube and then another. (Tsereteli, 1887: 1)

This was not merely a refreshing drink, but as a non-alcoholic *sociable* beverage, unencumbered by the rules of ritual consumption of wine expressing hospitality and status relationships, or as a drink associated with masculine plebeian sociabilities and hooliganism (like beer) (Manning, 2012b: 151, 207–9), it became a drink expressing egalitarian liberal modes of consumption, so that prominent Kutaisi aristocrat and poet Akaki Tsereteli penned a poem to greet the new drink, calling it ‘a rival of other drinks, of wine, of beer…’ (Sigua 1980: 9).

But as delicious as Laghidze's waters were and still are, more central to the liberal “civilizing mission” of the Kutaisi boulevard was the way it afforded and indeed promulgated new manners, new forms of comportment and public sociability, specifically, walking. Like the English public parks discussed by Patrick Joyce, the principal form of recreation it offered was “active, not passive, in the sense that the purpose was enjoyment to a purpose, namely the recreation of the self through recreation of the body” (Joyce, 2003: 220). The Boulevard was often glossed as a “walking garden”, a novel infrastructure for novel forms of public comportment of “stranger sociability” (Warner, 2002: 56–57), embodied in various forms of sociable walking which presupposed a positive, sociable relation with urban strangers, that of the *flaneur* (Manning, 2022)*,* the self-conscious status display of the aristocratic of bourgeois *promenade,* and what Patrick Joyce. (2003: 219) calls “nineteenth-century liberal walking”, “which emphasised a controllable private self that could not be completely of the city yet was very much in it” (Joyce, 2003: 219–220). Walking is also intrinsically sociable, in that increases the possibilities of meeting more persons than simply congregating: “The object of walking in the park was to see what Olmsted called ‘congregated human life’” (Joyce, 2003: 220). Liberal principles of gardening made the park into what Otter. (2007) calls a “liberal object”, objects which work by indirect rule–delegation to infrastructure–in which infrastructures indirectly inculcate liberal subjectivity. The cultivation of public parks cultivated human freedom, as nineteenth century American garden expert Andrew Jackson Downing puts it, “…Out of this common enjoyment of public grounds, by all classes, grows also a *social freedom*, and an easy and agreeable intercourse of all classes” (1853 [1848]: 141).

The Boulevard was a novel kind of entity, was differentiated from a garden in the traditional sense by its singular purpose, a shady place for walking, completely opposed to a traditional garden, a place to sit in the shade and drink alcoholic beverages like wine. Not everyone understood this purpose. The plebeian Tbilisi poet Skandarnova, in a long poem satirizing the pretensions to urbanity of another peripheral Georgian town, Telavi, singled out this town's so-called boulevard for especial contempt. As a Tbilisian urban poet whose ideal garden was primarily the Tbilisi gardens of Ortachala, a place for drunken festivity sitting in the shade under trees and not urbane strolling, this “boulevard” (a term unfamiliar to him and his readers, which he explains in a footnote as “walking garden”) seemed to Skandarnova as neither fish nor fowl, he couldn’t see what the point of it was or how it was even a garden:

Nor is your boulevard* [*bulvari*] so praiseworthy.

None of its four parts provide any shelter.^1^

It's neither a garden (*baghi*), nor a cottage garden (*baghcha*), nor a vineyard.

One won't profit there in the noonday sun.

* What Telavi's garden for walking (*saseirno baghi*) is called

(Skandarnova, 1879: 68)

The Kutaisi Boulevard was an aspirational infrastructure (Larkin. (2013: 332–334), it sought to turn sleepy old-fashioned Kutaisi into a modern European city. It also embodied a kind of civilizing mission, creating liberal publics, promulgating new forms of public sociability: a public park where one drank fizzy soft drinks and strolled in the shade, rather than sat down in the shade and got drunk on wine (as was the norm in plebeian gardens of Tbilisi favoured by Skandarnova (Manning, 2023)). The public boulevards of Georgia, starting in Kutaisi, and eventually spreading to peripheral towns like Telavi, were the paradigmatic sites for inculcating aspirational programs for liberal walking and strolling as the primary genre of “being in public”.

## The Boulevard as public

According to virtually any account from the 19^th^ to twentieth century, the Boulevard was the quintessential “public” space, explicitly compared to the Athenian Agora, the Roman Forum, the British Parliament. More than that, the Boulevard was the place that constituted the intelligentsia “public”: “If you want to see Imereti's ‘public’, come out on the “Gulvardi” a calm, pleasant, clear day…” (Nihil, 1894: 4). This public was something you didn’t have to imagine (a la Benedict Anderson, 1983), you could go out and see them on a clear day, on the Kutaisi Boulevard. The Boulevard was intimately connected to the idea of intelligentsia “public” both as face to face urban public created by the circulation of people and a more abstract space created by the circulation of texts. While much of the literature on publics (e.g., Warner, 2002) treats publics as almost exclusively a nonlocalized disembodied space created by the circulation of texts, all texts have to arise somewhere, be read somewhere, be discussed somewhere (Manning, 2022): in Kutaisi, and indeed much of Georgia, this place was the Boulevard. As Anderson himself notes, the public is partly imagined, and is partially inferred: When one reads a paper one imagines other readers doing the same in the disembodied public, but one also sees others reading newspapers in public spaces, parks, cafes, streetcars, and in those same places, one discusses what one has read with them:

At the same time, the newspaper reader, observing exact replicas of his own paper being consumed by his subway, barbershop, or residential neighbors, is continually reassured that the imagined world is visibly rooted in everyday life. (Anderson, 1983: 35–36)

So, for there to be textually mediated disembodied literary “publics,” there must *also* be specific times and places for people to talk, hang out, socialize, and discuss the news, producing face to face urban publics. This is particularly true of the nineteenth century (Anderson's model of the newspaper is decidedly late twentieth century), where the “news” was not evenly distributed over the distances of the nation, but was mostly produced and consumed locally, within the same city on the same day. For most of this paper I will treat “public” in this hybrid sense which accords with its use in Georgian intelligentsia discourse (see also Manning, 2012, 2022). The generation of Georgian intelligentsia writers who created the first Georgian newspaper, *Droeba* in the 1860s-1870s*,* and who constituted the first imagined community or “public” of Georgian intelligentsia as “Georgians, that is, readers of *Droeba*” (Manning, 2012, 2022), were almost all born or educated in Kutaisi, they spent their youth walking, sitting and talking on the Kutaisi Boulevard, a fact which they never failed to mention in their later writings. When they moved to Tbilisi and started the newspaper Droeba, they wrote feuilletons: The feuilleton, the prototypical genre of the newspaper, a genre which could only be found on a newspaper page, but could only be written in and about the streets and gardens of a city, was a fundamentally urban genre which was “tied together by the loose and whimsical transitions of a digressive persona wandering from topic to topic—and sometimes, in the conventional role of flaneur, from place to place as well” (Morson, 1981: 16). For these feuilletonists, urban gardens were public spaces of the city par excellence, and feuilletons presupposed an intimate shared awareness of this shared urban environment between writers and readers (Manning, 2022: 594). The feuilletons written by the Kutaisi intelligentsia in Kutaisi or Tbilisi always portrayed the concrete urban public as people walking and talking in urban gardens. When they compared the attractions of different gardens of the city, these gardens became metaphors for different kinds of newspaper feuilletons (Manning, 2022: 600–601, Manning, 2023: 4). The digressive structure of the feuilleton was like the meandering of a garden path or the meandering of a chat whilst walking in the garden (Manning, 2022: 594–601). One might say the Kutaisi Boulevard was the original embodied face to face “public” that informed the imagination of urban life and gardens and their imagined community of literary publics of their feuilletons.

## The Boulevard as multispecies plant public

According to the myth of the Boulevard, Georgian literary publics were born in the shady lanes of the Kutaisi Boulevard. What I intend to do here is attend to this exceptional urban garden in its history as what John Hartigan. (2015) calls a multispecies “plant public”, embodying not only a “purified” exclusively human sociological sphere but a “hybrid” heterogeneous more-than-human multispecies sphere (drawing on the familiar distinctions from Latour, 1993). Cultural multispecies assemblages like gardens and boulevards are “… are specifically constituted to carve out a domain of the nonhuman within urban spaces”, and this nonhuman domain paradoxically comes to be a central stage for human social action that he calls a “plant public” or “multispecies public”. Hartigan asks in what ways the very idea of a public, normally considered to be purely a relationship between humans, is in real cities actually anchored to multispecies assemblages like gardens (his example is botanical gardens, but the lessons are portable to other garden types). He says, “if publics are decidedly human…then how do we account for the presence of so many multispecies arrangements in their midst?” (2015: 484). Hartigan begins with the disembodied “print public” sense of public discussed above, a public “composed of self-reflexive readers hailed by various nationally mediated cultural forms”, but he also allows that “these sites are decidedly urban; as parks, they are key locales where publics assemble and constitute themselves,” that is, publics in the second sense. Lastly, he notes that gardens act reflexively on the urban environment as a kind of public “green infrastructure” “greening the city”, helping “people glimpse our entanglements with (and dependence upon) multispecies assemblages” (Hartigan, 2015: 484–5, for green infrastructures in Tbilisi see Gurchiani, 2025). These three dimensions of “public”, he argues, allows a way of rethinking urban human publics as “multispecies publics”.

As I will show, the Kutaisi Boulevard as a multispecies space touches on all these three dimensions of public: a densely mythologized intertextual space where the print publics of Georgia took form (as demonstrated by the many statues and plaques one encounters at every step today), but also the way that the Kutaisi Boulevard (unlike most of the other boulevards of Georgia) is referenced continuously becoming a textual topos as well as a place; secondly, as a concrete space where urban publics could, each and every day, go out and encounter each other as a face to face public, in person; and thirdly, as a multispecies public “green infrastructure” which might, or might not, exhibit desirable properties for human walkers and/or nonhuman visitors, or exhibit a stable or unstable mix of human and nonhuman actors, which might cause it to cease to act as an infrastructure for one, or the other, kind of actor. While the point of departure for this comparison is Kutaisi, at certain points I will be making a sustained comparison with another Georgian boulevard in the even more peripheral Eastern Georgian town of Telavi.

## The material semiotics of green

Kutaisi was in those days a green garden city: “somewhere between a city and a garden….If you were to see the city from afar, it would have seemed to you to be one big garden…” (Pirtskhalava, 1988: 15) and the Boulevard was the “central garden” of this garden city. The fact that it was green marked it as the central public space, as well as a *multispecies* public space. The colour green in cities “is a synechdoche for *landscape*” (Doherty, 2017: 5, original emphasis), the green nonhuman elements stand out as within the human city, as Joyce points out (2003: 223): “the park or garden is the “ultimate ‘other’ space in the city, the green, earthy, rural and natural heart of the built, crowded and man-made city.”

The emergent spatial rhetoric of what I will call “liberal gardening”, green spaces, gardens and boulevards, multispecies assemblages of humans and nonhumans, territorialize liberal ideas and practices of publicness. What Patrick Joyce calls “liberal walking” (Joyce, 2003: 219–221) in public space in general is descended from genres of walking in gardens. These liberal habits of walking, Maussian “techniques of the body” (see Meneley, 2019, 2025), were least partly inculcated by the “liberal gardening” of park designers like Olmsted, whom Joyce calls a “self-conscious designer of freedom” (Joyce, 2003: 220) (very different from the illiberal infrastructures constraining freedom of walkers discussed by Meneley, 2019, 2025). This “liberal choreography” by which designers of freedom like Olmsted imbued the garden paths with liberal constrained freedom (“engagement without compulsion” (Watelet, 2003: 27) is also a general feature of the “liberal” English landscape garden of the eighteenth century (Egbert, 2002, Sommer, 2007), which was also widely adopted elsewhere by Georgian aristocracy.

This role of green heterotopias to demarcate central public spaces is a relative novelty in the colonial city. In colonial period cities of Tbilisi and Kutaisi gardens moved from marking urban peripheries to marking the urban centre. Both these cities anchored their new urban public centres around public boulevards and public gardens, in effect completely inverting the received spatial logic of the city and the meaning of the colour green, where green spaces formed the suburban frame of an exclusively human city space (Walcher, 1997). Before, green represented a private walled retreat from public life; after, green territorialized publicness; places for new models of public comportment and presentation of self before an audience of strangers, liberal walking and flânerie, strolling in shady public spaces with fellow city-dwellers.

Tbilisi (and the Eastern provinces of Kartli-Kakheti) was conquered by the Russian empire in 1801 and building of the colonial city in the erstwhile suburbs begins around that time; Kutaisi and Imereti was conquered by Russia around 1810, and the Imeretian royal properties, including the gardens and spaces that would become the Boulevard, became property of the Russian colonial state around 1820. In Kutaisi, as in Tbilisi, these new walking gardens in the erstwhile suburbs became the public center of the new Russian “colonial” city so that the inherited opposition between human city centre and suburban gardens of the pre-colonial period becomes the familiar Orientalist colonial opposition between the winding streets of the old city and the straight European streets of the colonial city. As Michael Gilsenan argues, this colonial urban assemblage created an emergent

Cosmology of two separated worlds: One was the place of the irrational, secret, superstitious, dark and threatening, diseased, tightly packed native quarter; the other was open, linear, public, revealing, centred, rational, and insisting on its hierarchies—the space of power and status. (1982: 201)

The green space which residents of Kutaisi at that time called a “boulevard” was close to what we might now call a “public park”. This was an infrastructure for sociability, and most of all, for walking. The Kutaisi Boulevard notably was among the first public spaces in Georgia that featured what noted American landscape architect Frederick Law Olmsted would later call, in attempting to nail down the core meaning of the elusive term “park”, “park-purposes”, which he defined as a kind of hospitality to walkers:

[W]e may say that park-purposes means a purpose to make gracefully beautiful in combination with a purpose to make interesting and inviting, or hospitable by the offer of a succession of simple, natural pleasures as a result of easy movements. (Olmsted, 2015 [1868]: 436

Parks are hospitable to human publics. One might say that most of the infrastructural affordances of urban space that nurture urban publics amount to what Olmsted calls “hospitality”, which “consist[s] in conditions which make the ground appear pleasant to wander over” (2015 [1868]: 438). This hospitality appears to consist in two affordances: (1) that it should be amenable to pleasant strolling, an affordance embodied by the path, and (2) that it should have “hospitable scenery” (436), including the affordances of trees in framing and screening the park's visual field and providing shade for walkers (438), that is, the landscape. The hospitality of this green infrastructure is divided into two parts, a place for people, the path, and a place for plants, the landscape.

Olmsted's definition of “park-purposes” thus combines garden-as-landscape and garden-as-infrastructure. In a garden considered as landscape, it is the plant life that attracts attention; as an infrastructure, it is largely the condition of the usually ignored, presupposed, garden paths and sidewalks that do so. After all, the garden path forms the infrastructural substrate for the aesthetic enjoyment of the plants of the garden. Nor is the relationship between green and public in a green public space arbitrary, as early American garden theorist Downing argues, the green aesthetics of gardens draw the human population, high and low, there to mingle, so that the cultivation of plants cultivates, in turn, the cultivation of egalitarian human civilized society, gardening becomes nonhuman basis of the humanistic liberal theory of publics. In what could be a description of the affordances of the Kutaisi Boulevard, he says:

[If you] make the public parks or pleasure-grounds attractive by their lawns, fine trees, shady walks, and beautiful shrubs and flowers, by fine music, and the certainty of “meeting every body,” and you draw the whole moving population of the town there daily….. I see the public watering-places filled with all classes of society, partaking of the same pleasures, with as much zest as in any part of the world; and you must remember that there is no *forced* intercourse [interaction] in the daily reunions in a public garden or park. (Downing, 1853 [1848]: 143–143, original emphasis)

The oft-overlooked presence of the garden path within the green space brings us to the next point. These gardens were for Georgians a new kind of garden, a “walking garden”. The “walking garden” was so systematically different from the received Persianate garden form (*baghi*) in form and function that the residents of Kutaisi adopted a foreign term, “boulevard” (Balanchivadze, 1959), to suggest that that a garden for walking was, so to speak, a hybrid green space somewhere between a garden and a street. But it was also understood by virtually everyone to be a borrowed (and idiosyncratically applied) form, presumably from the French because of the French name (which admittedly has a different meaning) and geometric garden layout stereotypically associated with French, rather than English, gardens (Balanchivadze, 1987). As an alleged borrowing from a prestigious European source (after all, Persian gardens are also linear and geometric in form (Alemi, 1997, 2007, Walcher 1997)), this new green space in Kutaisi could serve as an aspirational emblem of modernity. As Larkin. (2013: 332–334) stresses, infrastructures are aspirational, materialized promises of progress and modernity, they can have affective and aesthetic values, “operating at the level of fantasy and desire” (Larkin, 2013: 333) that equal or exceed their technical properties.

In Georgia, as in much of Europe, trees, gardens, boulevards, and other “green objects” (Khmaladze, 1985: 53) have been treated since the nineteenth century as part of a comprehensive sanitary and hygienic green infrastructures for the mental and physical well-being of the residents, human and nonhuman. Accordingly, critiques of the failures of modernity in Georgia usually foregrounded absent or failing infrastructures, including green infrastructures like boulevards. For example, discussion of green infrastructures in Tbilisi have since the nineteenth century dwelt upon the insufficiency of green spaces in Tbilisi for the population ((*Iveria* 1878 2.13, pp.1–3, Chkhetia, 1940: 192–4, Khmaladze, 1985: 50–53, Patarkalashvili, 2015: 79, Patarkalashvili, 2017: 187–8, Gurchiani, 2025: 5–6). In peripheral cities like Kutaisi or the even more peripheral East Georgian town of Telavi, which did have green spaces in relative plenty like Boulevards, critiques of green infrastructures often attended not to the *absence of green* (as in Tbilisi) but to the state of the by turns dust or muddy garden path which made the boulevard no longer adequate for walking.

Lastly, gardens, “Plant Publics” (Hartigan, 2015) overflow their human addresses, and become “multispecies infrastructures” hospitable to both humans and other beings: “Though human politics and values are surely embedded in infrastructures, once infrastructure becomes a source of nutrition for trees, the values and politics inscribed on the urban landscape ceases to be wholly human oriented” (Jensen, 2017: 10). As Jensen notes, the other-than-human denizens of these multispecies publics or multispecies infrastructures thus threaten to decenter the aesthetics and politics of infrastructures from the human.

Parks, like the sewage pipes explored by Jensen. (2017), are hospitable to “multispecies publics”. Following Jensen by drawing inspiration from Von Uexküll's concept of the *Umwelt* or lifeworld of different organisms, these green infrastructures form the *Umwelten* or aesthetic and practical lifeworlds of what we can call a “multispecies public”, something that can be the object of a more-than-human ecological aesthetics. Indeed, gardens and forests are so obviously examples of multispecies infrastructures that Von Uexküll used both the garden and the forest to show how a single greenspace could be a landscape/infrastructure adaptable to the *Umwelten* of a multispecies public (Von Uexküll, 2010: 114–5). His remarks about the forest can be extended to the hospitality of any garden space (it is noteworthy that he never seems to consider the plants themselves as more than a context for human or nonhuman *Umwelten*):

Are we not taught by this example that the forest, for instance, which the poets praise as the most beautiful place of sojourn for human beings, is in no way grasped in its full meaning if we relate it only to ourselves? . . . The meaning of the forest is multiplied a thousandfold if its relationships are extended to animals, and not only limited to human beings. (2010: 93).

So, moving beyond the humans and the plants, parks are hospitable to a number of nonhuman beings, birds and other animals. But this tendency of the hospitality of gardens to overflow the boundaries of the human could, from a humanistic perspective, be itself a sign of failure of modernization, becoming a topic of concern for the modernist satirical critiques of infrastructure in peripheral cities like Kutaisi (in West Georgia) and Telavi (in East Georgia), as a sign of Kutaisi's or Telavi's inability to form properly modern, that is, primarily human, public spaces.

## Boulevard is a place for walking

**“**The city [Kutaisi's] garden, which locals call the “Boulevard” (*bulvari*), is the only place for walking (*saseirno adgili*).” (K.-me, 1903: 2).

Since it is so obviously a garden, why was it called a boulevard? The French term boulevard was adopted because it was a new kind of garden, specifically “a walking (boulevard) garden”” (*saseirno (bulvari) baghi)*)) (Sasoplo Gazeti, 1912), different from the royal gardens it replaced, as Balanchivadze stresses:

In old Georgian the term garden [*baghi*] referred to a place where fruit trees were planted, but a garden which was set aside for spending time, where were planted trees pleasing to the eye, that is, they were not fruit trees, but decorative trees, was called a *“*paradise” [*samotkhe*]. So, the history of today's central urban garden begins in the 1840s. It was built as a place of rest and relaxation and not as a garden of fruit trees. And that's why among other things, it was not called a “garden” [*baghi*], but a “boulevard” [*bulvardi*], which was borrowed from a foreign language and which [originally] means a wide street for strolling shaded by trees. (Balanchivadze, 1959: 1)

No one ever mentions the Kutaisi Boulevard without mentioning that the word is at best an idiosyncratic application of a foreign term to a novel phenomenon. Because the term is used in Tbilisi in its normal sense, the resulting terminological confusion is considerable, but I would argue this confusion is key to understanding the Boulevard as a novel kind of garden. Outside of Tbilisi, this new Georgian understanding of boulevard began to spread and mutate (and new boulevards crop up in Georgia to this day). The Eastern city of Telavi soon had a boulevard modeled on the Kutaisi Boulevard by 1879, and other cities (Batumi, Zugdidi, Sukhumi, Poti) followed. Today new boulevard in this idiosyncratic Georgian sense are still being created.

Meanwhile, in the capital Tbilisi, the term was closer to the received meaning as a kind of street, but still could be systematically ambiguous. For writers like Ilia Ch’avch’avadze writing in the nineteenth century (Iveria 1878 2.13, pp.1–3), writing about the general absence of gardens as green infrastructures necessary for human health and happiness in Tbilisi (and arguing about many places, including cemeteries, that could be converted into gardens (Gurchiani, 2025: 5–6)), the term *bulvari* (boulevard) might be used on the very same page as a kind of street, that is, a place unsuitable for children to play, or as a kind of garden, as part of a series of green infrastructures (“places for entertainment” *saseiro adgilebi*) alongside *baghi “*garden”, *khevnari* “place with trees”, *kheivani “*tree-lined *allee*”, *ch’ala* “riverside grove”. From the context, it's impossible to guess what kind of *bulvari* he was imagining in each case.

More generally, the term boulevard was sometimes used for the two large tree-lined central streets on either side of the Kura river (Golovin and Mikhailov Prospect). For French writers like Floriant Gille. (1859: 267), Tbilisi's Golovin Prospect was transparently a “completely European boulevard” which was the centrepiece of “New Tbilisi” as an outpost of Europe standing in contrast with the winding streets of the “Oriental” Bazaar and the old city (cf. Gilsenan, 1982):

The old Tiflis in a word, is still Asia; but in the last twenty-five years the new Tiflis has taken on a European appearance, with its long boulevard, its spacious and elegant houses and its rich shops. (Gille, 1859: 262)

Sometimes, however, for Georgians the term boulevard in Tbilisi meant only the shady tree-lined *allee* (usually called a *kheivani*) for walking on either side of the street (Chkhetia, 1940: 181, 185). Either way, these kind of boulevards are what I will call hybrid “Boulevard-streets”. Since, as in Europe, central walking gardens are often appended to boulevard-streets, in Tbilisi the hybrid of the two as a shady place for walking was a garden-and-boulevard (*baghi-bulvari*) (Barnovi, 1961 [1909]: volume 2: 155).

The term “boulevard” in contemporary Georgian dictionaries shows the same over-all ambivalence. The Georgian *Dictionary of Foreign Words* gives the “Tbilisi” definition: “*Bulvari*. Fr. Boulevard. A wide tree-lined alley [*kheivani*] along a city street [*kucha*] (usually, in the middle).”^2^ The second definition from the Explanatory Dictionary (www.ganmarteba.ge) gives it the “Kutaisi” definition as “a place for strolling or resting with tree-lined alleys (*kheivani*), gardens (*baghi*) and pedestrian footpaths.”^3^ Behind all this referential confusion, the Kutaisi “boulevard-garden” shared with the Tbilisi “boulevard-street” two things: (1) formally, a boulevard in either sense incorporated a *kheivani* (straight tree-lined paths), a term borrowed from the Persian *khīyābān* (Alemi, 1997, 2007) which originally means a shady, straight tree-lined path in a garden; (2) functionally, it was a space suitable for walking, strolling, promenading. The basic infrastructural unit that is shared between the two kinds of “boulevard” is the *kheivani* (rarely glossed as French *allee,* Russian *alea*) consisting of a straight garden path shaded by rows of trees: the garden *path* is as important as the trees that shade it.

As a result of the fact that a French term (*bulvari*) was borrowed to replace an originally Persian one (*baghi*), the dyad *bulvari* vs. *baghi* seemed to express an orientalist cosmology of Western versus Eastern garden traditions (cf. Gilsenan), but the reality is more ambiguous than that. If Georgian “boulevards” bear a European name that places streets and gardens on a kind of natureculture continuum, underlying this European term applied to diverse referents is a Persianate term (and central element of garden design) in Georgian, *kheivani.* What all the “boulevard-gardens” in Georgia have in common is that they are fenced, quadatric in shape, and consist of a number of parallel straight tree-lined walks (using the Persian-derived term *kheivani,* which is standard, more rarely the French derived *allei* (*allee*), in Russian texts *alea*), lined with shady trees and flower beds, sometimes intersected by another set of *kheivanis*, with decorative elements like fountains or pavilions set at the intersection of these *kheivanis* (Balanchivadze, 1859). While the overall geometric symmetry of the boulevard form, as well as its name, strikes many commentators as a sign that it must have been designed by some unknown French gardener (Balanchivadze, 1987), this design (and terminology of components like *kheivani*) is absolutely typical of the Persian *chahar-bagh* style garden (Alemi, 1997, 2007), only with visually permeable fences rather than high garden walls and no fruit trees. Indeed, while French gardens are symmetric, they often involve non-parallel intersecting geometric lines and paths without shade trees (for example, the nineteenth century Zugdidi Botanical garden is laid out in a French pattern of this kind). The Kutaisi Boulevard-garden oscillates undecidably in a virtual state between Western (French) and Eastern (Persian) hegemonic garden traditions.

Like a Persian *Chahar-Bagh* (Alemi 1997; Walcher, 1997), a boulevard is constructed exhaustively out of a series of *kheivanis.* Most Georgians I know give this term a Georgian folk etymology, since it seems to have something to do with trees (*khe*). Thus 17^th^-eighteenth century Sulkhan-Saba Orbeliani dictionary defines *khevani* (= *kheivani*) as “trees standing in a row” (Orbeliani, 1949: 901), not even mentioning the path, as if it were etymologically related to the dictionary-adjacent word *khevnari “*a place with trees”. But the term is instead derived from the Persian garden term *khīyābān: “*In an enclosed garden, a *khīyābān* is a promenade forming a straight alley under the shade of trees with a water canal in the center” (Alemi, 2007:115). Unlike Persians, for whom kheivani eventually moved from an element of garden design to urban design (Alemi, 1997, 2007, Walcher, 1997), coming to mean a long straight treelined street, a boulevard (Naraghi, 2023: 148), Georgians never quite adopted the term *kheivani* to mean a kind of street, but certain kinds of large streets (boulevards or prospects) are composed of streets flanked by *kheivanis*, and certain kinds of walking gardens (also boulevards, as opposed to gardens (*baghi*)), flanked by city streets, are also composed of a number of *kheivanis*. What we will see is that the state of the *path* as a component of the *kheivani* becomes crucial for assessing the “hospitality” of this green infrastructure.

## Aspiration and abjection: The state of the garden path

Georgian accounts exulted about the beauty of their Boulevard with its (now French) *allees*:

The first thing a person sees when they enter Kutaisi is Kutaisi's beautiful and beautifully decorated Boulevard. My God, what beautiful lime trees and other shady trees line this garden*,* what breezes are in its *allees* [French, transcribed in Georgian as *allei*]*,* what coolness in their shade!… (Skandeli [Niko Nikoladze] 1871: 1)

However, foreigners writing around the same time could be somewhat jaundiced, even savage, in their assessment of the achieved material reality of the mythic Kutaisi Boulevard:

The public garden at Kutais is a plot of ground the size of a large London Square, with walks down the middle, and a few trees, but no flowers; it is, in fact, like an unkempt piece of Regent's Park*.* (Freshfield, 1869: 87)

But most discussions of the Boulevards in Kutaisi and Telavi (the two earliest boulevards) did not usually dwell too much on the aesthetic landscape of the park, the plants and flowers, but rather focused on the (often rather abject) state of the garden *paths* as infrastructures, specifically, for walking. There is a tendency to think of gardens as belonging to an other-than-human world of aestheticized landscapes rather than the other-than-human world of infrastructures. But a walking garden, and a fortiori almost any garden, is nothing without the often seen-but-unnoticed walking infrastructure of a garden path. In the same way, the principal constitutive element of a boulevard is, as we saw above, a set of tree-lined paths, here called *allees,* or *walks.* While one might, as in the quote above, attend to the shade afford by the trees, the path was often found to be in a condition not suitable for walking.

The condition of the garden path takes us away from the beauty of the plants (the landscape) to the human walkers (the infrastructure). The problem, briefly, is that walking is a destructive activity, walking destroys plant life (hence the signs that say “stay on the path, stay off the grass”), leaving the path as a site of dirt. In the summer, in the absence of frequently neglected watering, this dirt turns into clouds of dust (and in the rain, a lot of mud). The central problem of walking on paths that are neither paved nor covered in broken up brick is that they are by turns very dusty or very muddy. In Kutaisi, for example, because of the pervasive dust on the garden paths, walkers would avoid the dusty garden paths and stick to the paved sidewalks that surrounded the garden, or the southern side of the boulevard where there was pavement (k-me, 1903: 2) (the external margins of the garden which were called *baghis k’ide “*the edge of the garden” above). Most accounts of boulevards do not exult about the beauty of the landscape, but instead find fault with the infrastructure: the failure of each city to thus prepare the paths with crushed brick or at least water them to reduce the dust is a perennial critique, part of a more general intelligentsia taste for what is often called “critical realism”, that is, finding fault with the abject state of public infrastructures. So here, as with other infrastructures, we find these otherwise invisible infrastructures become the most remarked upon aspect of the gardens when they fail, when they move from aspirational “sublime” infrastructures to degraded, abject “picturesque” infrastructures.

Dust and mud is an attendant problem of other walking garden “boulevards”. For example the peripheral western Georgian city of Telavi, a city that apparently once had a Kutaisi-style boulevard, that Tbilisi poet Skandarnova thought was ridiculous. A town like Telavi had only two major public places, both of them gardens, or rather, walking gardens: one was the boulevard and the other a little hill (Nadik′vari, which is still a walking garden today) (Moagarak’e, 1903: 3, Dimitrashvili, 1904: 3). A visitor to Telavi didn’t have much to talk about because there wasn’t much to do, so the attention of visitors was often drawn to these two places, as places for congregating and especially for walking, a fashionable activity then as now. As in Kutaisi, remarks on the Telavi Boulevard often consider not its aesthetics as a garden, but its failings as an infrastructure for walking. A “letter from Telavi” in 1903 begins with a consideration of the importance of a boulevard kept in proper condition for walking or strolling (*seirnoba*):

I think that if the people who live here and particularly those who like walking were aware of the importance of walking, they would not treat the Boulevard as they do now. I wish I knew how the pedestrians feel when they inhale dust from women's dresses, because the alleys are not watered. (Moagarak’e, 1903: 3)

Another visitor reports that while Telavi has many places affording a lovely stroll, that nevertheless the centrality of the Boulevard makes it a “base for walking”, and whatever else the caretakers did to beautify it, in the absence of regular watering, dust became a perennial problem.

Walkers reject all these other places [for walking such as Nadik′vari] and only mount an attack on the Boulevard. This, first of all, is closer and second is also close to the clubhouse to boot, where there is billiards and a restaurant. They stroll at morning, mid-day, evening and night. Because the walkers have turned the Boulevard into a base for walking, the caretakers of the garden also strengthened their position. The beautiful grass was mowed and they fenced it off with barbed wire. The walkers got used to everything, they only complained that “we had to inhale so much dust”. (Dimitrashvili, 1904: 3)

Walking gardens, considered as infrastructures, draw attention to the way the shade offered by the trees and the aesthetic effect of the growing green things affords not only a space of encounter between humans and nonhumans (plants) but also more centrally a social space of encounter in public between humans. However, as infrastructures for walking, the focus quickly moves to the less aesthetically salient importance of affording these gardens with walkable paths. In this sense, walking gardens are only commented upon when they fail to act as invisible, working infrastructures.

## Interzoological walking on the Boulevard

**Figure 4.**
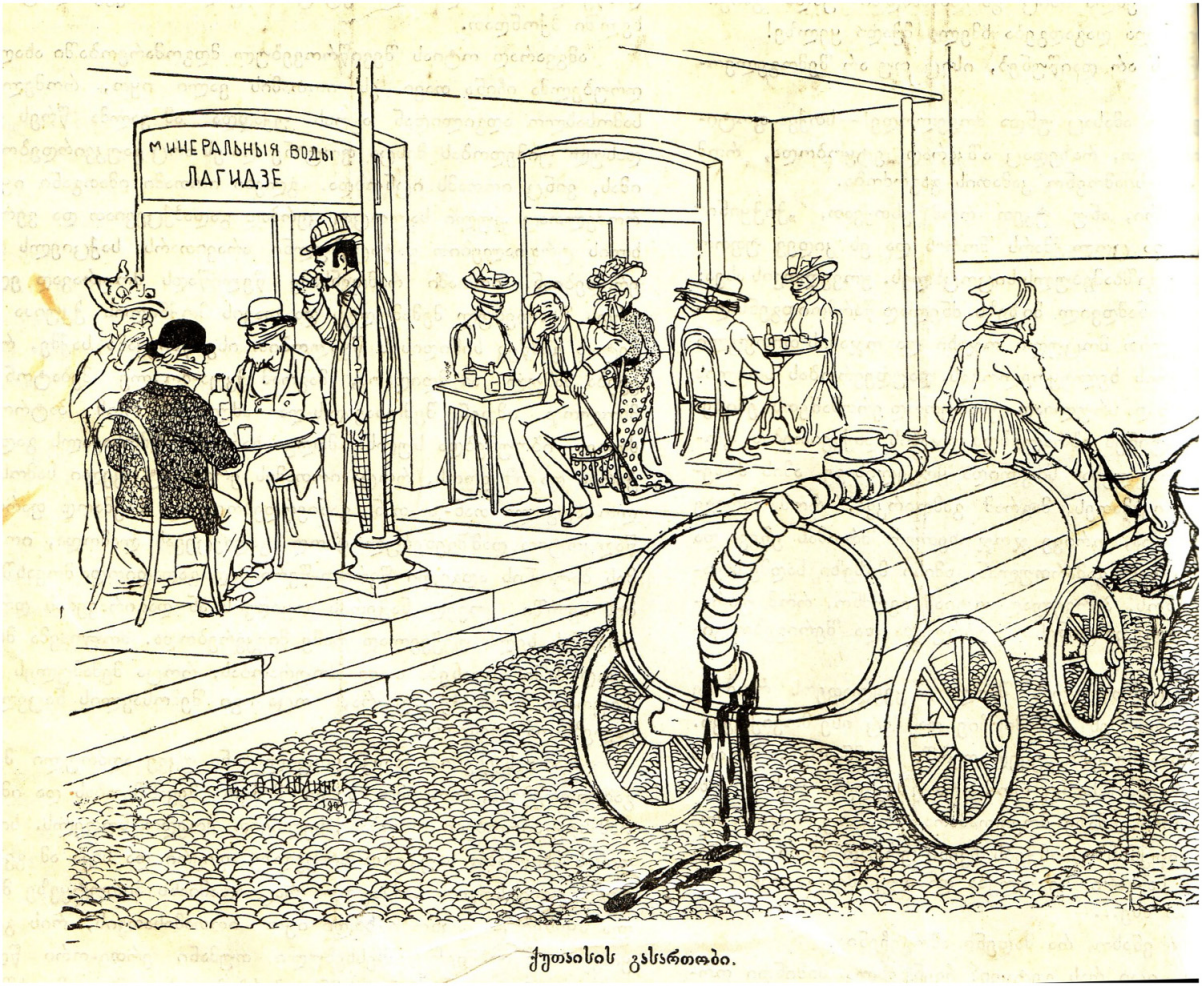
“Kutaisi entertainment” (the sign above the café door reads [in Russian] “Laghidze Mineral Waters”) (Schmerling 1903: 4).

Georgians themselves often abjected their own problematic attempts at achieving European modernity in satirical images that juxtaposed aspirational ideals of modernity, usually embodied in a public infrastructure, with the painful backwardness of the real achieved enactment of these ideal forms in local material terms. For example the Laghidze café mentioned above was an emblem of Kutaisi's aspirations to modernity closely allied to the Boulevard, since it began inside the Kutaisi Boulevard, and then moved across the street from it, and was the first establishment in Kutaisi that had electric lighting (a by-product of the need for ice for the fizzy lemonades, which were known at the time as “Artificial Mineral Waters”). In satirical images, however, this literal beacon of aspirational modernity is frequently mobilized instead as a ready-to-hand symbol epitomizing abjection. For example, what better way to display the lack of sewage systems in Kutaisi than to show Laghidze's café, by the Boulevard, with the genteel members of Kutaisi society seated there decorously covering their noses because of leaking waste cart on the street (Figure 4**)**?

**Figure 5.**
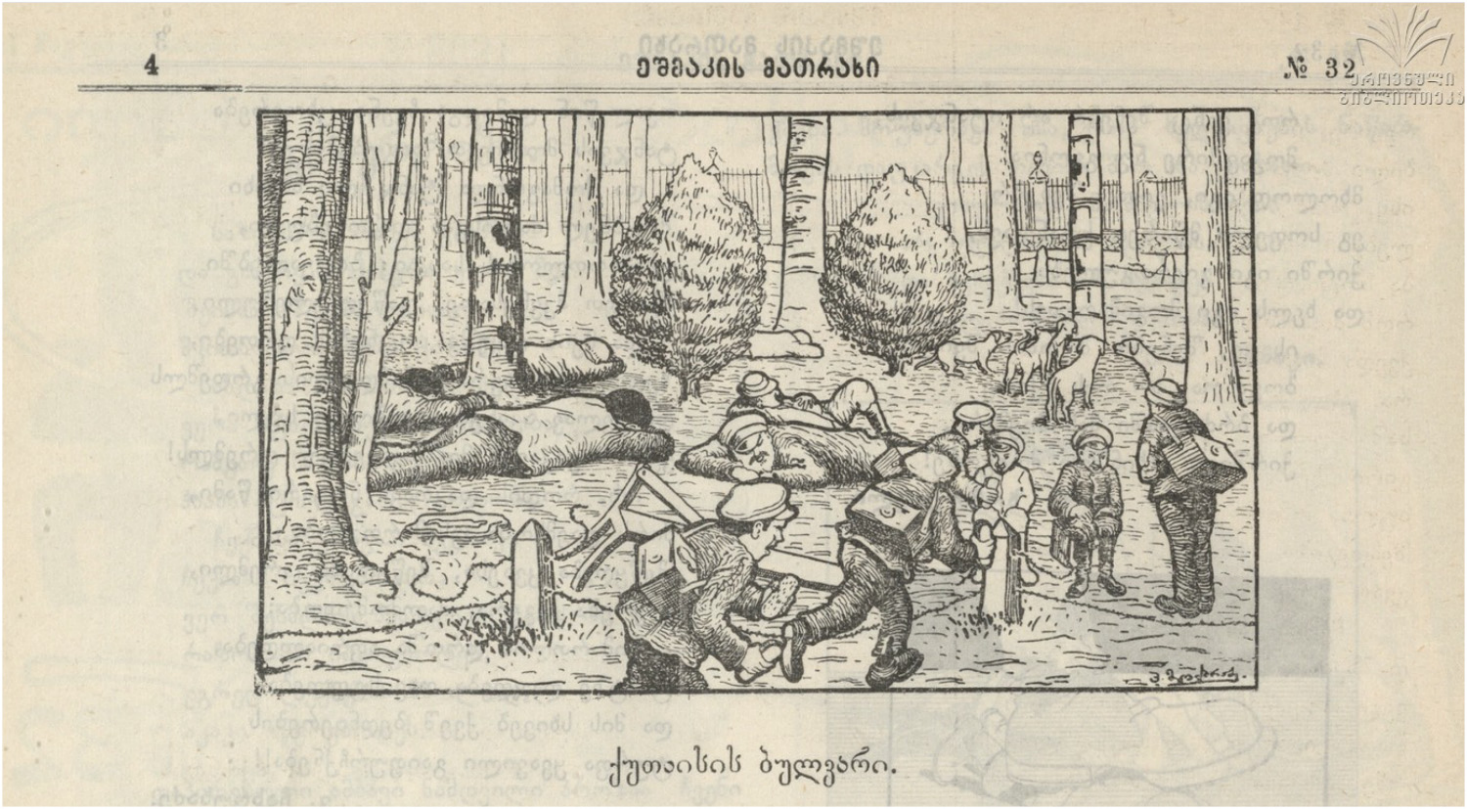
“Kutaisi's Boulevard” (Mgherabadze 1908: 4).

If the threat does not come from sewage carts on the adjacent street, the interior of the Boulevard is also shown as a space under a kind of multispecies attack: on the human side, vagrants and assorted rough sleepers using the public space in a disorderly manner; on the nonhuman side, what appears to be packs of feral dogs doing much the same (Figure 5 Mgherabadze, 1908: 4)

Like Kutaisi's Boulevard, Telavi's Boulevard embodied a perhaps even more precarious claim to be a hotbed of urban civilization as a small urban centre. As with any Georgian city, the Telavi Boulevard was the first thing any visitor to the city would mention: to epitomize or summarize the social and intellectual life of any Georgian city in the nineteenth century, a traveler or feuilletonist only had to check the pulse of the local boulevard. But even as it formed the centre of urban public life as a place for gathering and strolling, it also attracted satire, since it provided a handy image to judge the gulf between aspirations for modernity and the abject failure to achieve it.

**Figure 6.**
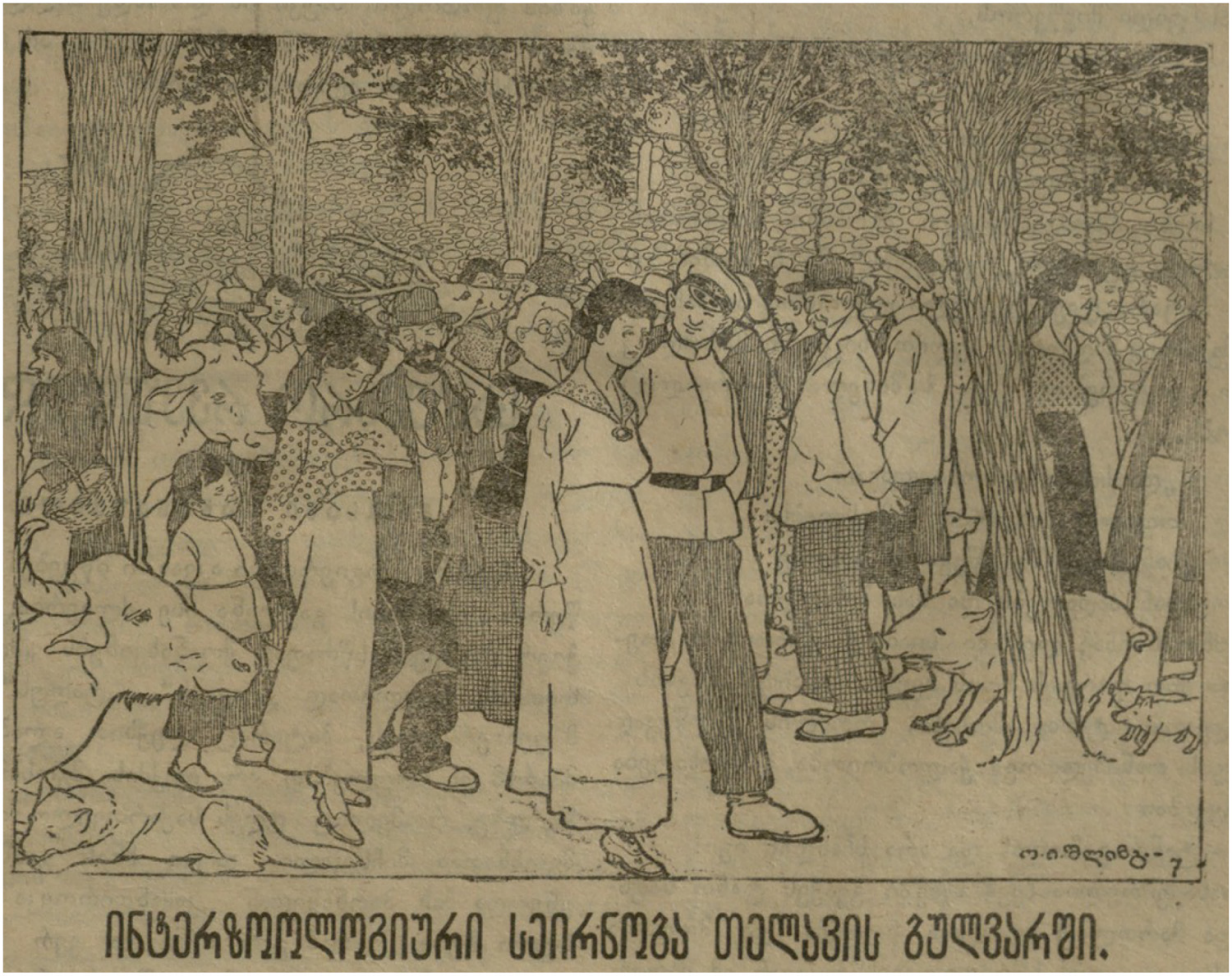
“Interzoological walking on the Telavi Boulevard” (O. I. Shling. [Oscar Schmerling] 1916: 1).

We have very few images, illustrative or photographic, of this boulevard. However, the one satirical image we have of this boulevard (Figure 6), “interzoological strolling on Telavi's Boulevard”, echoes the predicament of Kutaisi in broad terms, but instead of strollers being assaulted by the effluvia of human waste products, instead it is “interzoological strolling” where the same paths are shared with several species of farm animals as well as some improbable human types to add to the general chaotic mixture (an apparently Japanese woman is shown in the foreground left surrounded by cows, geese, feral dogs, and a stag). If gardens and boulevards are multispecies assemblages, including plants, perhaps well-behaved birds, and humans, such multispecies assemblages become destabilized by other zoological elements, packs of feral dogs or angry birds on the Kutaisi boulevard, or here, various farm animals, turning an urban space for strolling into a farmer's yard, perhaps implying that Telavi is not really able to sustain a distinction between city and village, and thence, between walking garden and household garden.

This cartoon satirizes the inability of Telavi to maintain the Boulevard as a public place for strolling for *humans.* But as a garden, the Boulevard is addressed to a multispecies public of humans and nonhumans, and comprises a kind of multispecies infrastructure. Gardens afford places of rest, relaxation, food and homes for many nonhuman animals, particularly birds, a presence that can be aestheticized alongside the shady trees and flowers. But in such gardens of order, neither planty nor animal agency that overflows the bounds of pure spectacle can be a source of enjoyment (see Power, 2005 on the aesthetics of nonhuman agency in “human″ and “hybrid” gardens). A Tbilisi poet like Skandarnova, who, as noted, could not see the point of a boulevard garden, with no shady places to sit and drink, would celebrate the multispecies relational world of Tbilisi gardens which embodied an almost cosmological (Sufi) animating principle of love (*eshkhi,* from Persian *‘ishq)* blurred the boundaries between humans and nonhumans, drunks and birds (on the Sufi cosmology of Tbilisi garden poetry see Manning, 2023).

When I had it all done by myself [i.e., arranged my garden],

Many visited me to see it!

They fell ill smelling the fragrance,

And were changed into flirts, slaves of the nightingale!

(Skandarnova, 1914: 15; translation by Davit Toklikishvili.)

But, at the same time, for rather more Europeanized liberal intelligentsia of Tbilisi, Kutaisi and yes, Telavi, this blurred world of this Persianate Sufi garden poetry, where drunken humans and feathered nonhumans exchange properties, could only represent a scandalous multispecies “poetic chaos” (Manning, 2023). For such new Europeanized elites, failure to maintain these boundaries between human and nonhuman, whether poetry addressed to print publics or face to face publics of boulevards, was scandalous, even a sign of abjection, of failure to modernize, in a country on the periphery of Europe which could not afford to recognize its ties to the Persian world nor, indeed, the nonhuman world.

## Talking about infrastructure with birds on the Boulevard

**Figure 7.**
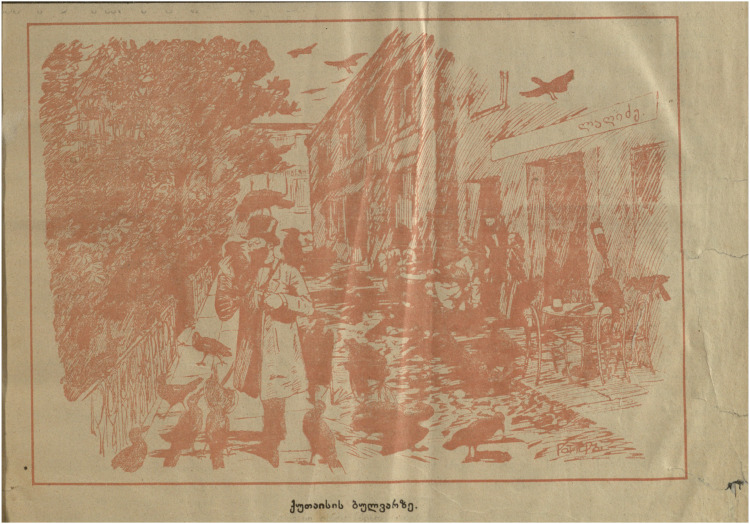
“On the Kutaisi Boulevard” (*Shuamavali* 1907. 22 July. No. 2 page 1).

I close with one last example of the Kutaisi Boulevard imagined as a multispecies public, this time a fantastic one in which animals lecture humans about infrastructure. It begins seemingly with another image (from 1907 entitled “on the Kutaisi Boulevard” (Figure 7)) where the precariously stabilized assemblage, the liminal space of the “edge of the garden” mentioned above between Kutaisi Boulevard and the Laghidze café across the street, which embodied Kutaisi's claims to urban civilization, is destabilized in yet another satirical cartoon. Here again, we find this beacon of civilization under attack by uncivilized, indeed nonhuman, forces. In an image reminiscent of Hitchcock's horror film *The Birds,* we see a murder of aggressive crows who seem to be attacking the café (here labeled in Georgian *Laghidze*).

The last cartoon seems at first glance to be a stand-alone cartoon of the previous satirical series, where the Boulevard/cafe assemblage (Civilization) are shown to be under attack variously drunken hooligans, farm animals, wild dogs, leaking waste carts, and finally birds. But the cartoon here illustrates an attendant satirical Aesopian piece, a *feuilleton* which mixes fact with the fantastic (Manning, 2022: 602–3). The respectable top-hat wearing man in the illustration turns out to be the head of local government, who is confronted by a murder of talking crows who turn out not to have come to disrupt the public order, but to express neighbourly concerns about the disastrous state of Kutaisi's modern infrastructures, or rather, the absence of them: lack of water, dusty streets and paths resulting from lack of water, mud when it rains resulting from inability to control the dust, and finally the absence of any form of public transportation (Ireteli, 1907:3).

This turns out to be an Aesopian fable (Manning, 2012a:41–5), a popular form of critique in which critiques of the state become more palatable when voiced by fantastic speaking nonhuman animals. This begins with the lead crow, perched on the man's hat, instructing him that the crows have assembled for a discussion, in accordance with the laws of the winged kingdom guaranteeing freedom of speech and assembly. The man tells them that regardless of whatever alleged laws they have, when they are in the territory of humans, any such meeting is forbidden as being in violation of local ordinance (paragraph 87) against public disorder, specifically the disorder represented by animals intruding into the human space of the Boulevard (the very thing satirized in the Telavi “interzoological strolling” cartoon). The laughing Crow leader says that they are not afraid of any civic rules or “87”, because they have gathered here for a discussion. The man wants to know who tasked them with this meeting and they reply:

Lead Crow: No one tasked us….We see in what circumstances are our neighbour bipedal animals and we decided, that since they cannot speak up, we should stand up for them.

The man wants to know what it could possibly be that the crows want to talk about. Inverting the original presupposition of the man and all the previous cartoons too, that animals themselves in orderly urban spaces that would be the sign of urban disorder, they say that they are concerned about the disorder in the city, specifically, the absence of critical infrastructures for humans (and, it should be added, crows) specifically water for drinking and other purposes. Absurdly, Laghidze's soft drink factory, the only reasonably modern establishment in Kutaisi, is invoked as a cure-all for the missing water.

Crow: Where is the water?

Man: In the Rioni river [which passes through Kutaisi]

Crow (laughing): Bravo!…. Everyone knows that the Rioni is water. I am asking you where is the water which Kutaisi residents can drink without it killing them.

Man: In the Laghidze's [soft drink] factory.

….

Crow: What an answer! In the Laghidze's factory there is sweet water. Is it really possible you intend to bring this water with pipes to rinse the streets?

Man: Why not, if you have the funds? If we were to rinse the streets with Laghidze's water, then nothing would be better than coming to Kutaisi, such a vanilla scent would be in the streets…..

Crow: Dimwit! We have visited almost every city. We have been to Tbilisi. In that city waterpipes have been laid, from which clean water is given to the inhabitants. We are asking you about this kind of water.

Man: (placing a finger on his forehead)… I have not forgotten, of course, we also have such water.

Crows: (together) You lie! You lie!

Main Crow: It's true you lie. Well where is such water? A whole day we are wandering about with dry throats and we haven’t found a single drop of water anywhere. Who drinks the dirty water of the Rioni?

Man: You are getting mad for no reason. If you don’t believe come over to the city office and I will show you, that we have such water.

Crows: (together) You lie! You lie! We already went there, but there isn’t any water….

Man: If you were in the office, sirs, perhaps you did not enter the archives. There had you entered you would have found in some old papers a project for bringing water.

In this Aesopian display of critical realism, the crows’ neighbourly interspecies concern for Kutaisi's failed modernity moves from one missing infrastructure to another, radiating outward from the Boulevard, and of course, Laghidze's soft drink factory/café (one crow is shown attempting to drink from a Laghidze's bottle): water for drinking and cleaning the streets, the dust in the streets, the mud, the absence of a streetcar system.

The Aesopian fable confronts the Crows’ practical interest in actually existing infrastructures, such as are found in Tbilisi, with unrealized plans and projects for creating such infrastructures that are preserved in old books in the archives in Kutaisi, which the man treats as being, in effect, the same thing as having water to drink right now. The cartoon looks like a series of others that present the same dilemmas of a failed aspirational modernity, in which, as the man notes, the crows are engaged in a form of disorderly assembly, specifically prohibited by local ordinance paragraph 87. But the story inverts this narrative, turning critical realism into a fantastic Aesopian feuilleton, where nonhuman voices of crows are critical realists, saying, as animals as in any Aesopian fable, what humans are not allowed to say. In fact the crows say this precisely. The dialog inverts the expectations set up by the cartoon, for the crows have not come to disrupt the human order of the Boulevard, but to improve it: fantastic Aesopian speakers, the crows, a nonhuman intelligentsia, always deliver a cogent critique of the existing reality, while the nonfantastic human speaker always replies with ridiculous, fantastic solutions referencing the existence of an as yet perhaps never-to-be realized project for the improvement of Kutaisi to be found in some old dusty state archives.

## Highlights

- Urban public gardens are addressed (albeit differently) to “multispecies publics”, including humans and nonhumans
- Urban public gardens are both aesthetic objects and infrastructures for sociability
- Gardens can demarcate the centres of new urban systems and territorialize face to face publics
- As infrastructures, gardens can both represent aspirations for modernity and represent abject failures to modernize
- The fact that gardens are multispecies spaces can represent a form of abjection, a failure to modernize

***

## Notes

1. “four parts” is not so much a statement of garden organization as a reference to the Persian *chaharbagh* “four part garden”, in which the four parts are largely symbolic of totality (Alemi 1997:75)

2.
http://www.nplg.gov.ge/gwdict/index.php?a=term&d=3&t=7831

3. https://www.ganmarteba.ge/word/ბულვარი

## References

[bibr1-25148486251371535] Alemi M (1997) The royal gardens of the Safavid period: Types and models. In: Petruccioli A (eds) Gardens in the Time of the Great Muslim Empires. Leiden: Brill, 72–96.

[bibr2-25148486251371535] Alemi M (2007) Princely Safavid gardens: Stage for rituals of imperial display and political legitimacy. In: Middle East Garden Traditions: Unity and Diversity: Questions, Methods, and Resources in a Multicultural Perspective. Washington, DC: Dumbarton Oaks Research Library and Collection, 113–137.

[bibr3-25148486251371535] Anderson B (1983) Imagined Communities: Reflections on the Origin and Spread of Nationalism. London: Verso Books.

[bibr4-25148486251371535] Balanchivadze N (1959) Bulvardi [Boulevard]’. *Stalineli*, 29 November: 1.

[bibr5-25148486251371535] Balanchivadze N (1987) Chveni Bulvari. *Kutaisi* 1987, no. 27 January: 1.

[bibr6-25148486251371535] Barnovi V (1961 [1909]) T'k’bili duduk'i. In: Barnovi V (eds) Tkhzulebata Sruli K’rebuli. Tbilisi: Sak SSR Metsnierebata Ak’ademia, 2. 150–171.

[bibr7-25148486251371535] Chkhetia S (1940) T'pilisis ist’oriidam. Enimkis Moambe V-VI, 177–207.

[bibr8-25148486251371535] Dimitrashvili . (1904) Telavi. Tsnobis Purtseli 1904(2541): 3.

[bibr9-25148486251371535] Doherty G (2017) Paradoxes of Green: Landscapes of a City-state. Berkeley: Univ of California Press.

[bibr10-25148486251371535] Downing AJ (1853 [1848]) A talk about public parks and gardens. In: Downing, Rural Essays. New York: George P. Putman and Company, 138–146.

[bibr11-25148486251371535] Dubois de Montpéreux F (1843a) Voyage Au Caucase*.* Tome I. Paris: Librarie de Gide.

[bibr12-25148486251371535] Egbert M-L (2002) Patriotic islands: The politics of the English landscape garden. EESE 5 https://webdoc.sub.gwdg.de/edoc/ia/eese/artic22/egbert/egbert.html

[bibr13-25148486251371535] Freshfield D (1869) Travels in the Central Caucasus and Bashan. London: Longmans, Green and Co.

[bibr14-25148486251371535] Gille F (1859) Lettres sur le Caucase et la Crimée. Paris: Gide.

[bibr15-25148486251371535] Gilsenan M (1982) Recognizing Islam. New York: Pantheon Books.

[bibr16-25148486251371535] Gurchiani K (2025) The many lives of the dead: What cemeteries in Tbilisi do. History and Anthropology Online 05 May 2025: 1–21.

[bibr17-25148486251371535] Hartigan J (2015) Plant publics: Multispecies relating in Spanish botanical gardens. Anthropological Quarterly 88(2): 481–507.

[bibr18-25148486251371535] Ireteli . (1907) Kutaisis bulvarze. Shuamavali 1907(2): 1–3

[bibr19-25148486251371535] Jensen CB (2017) The *umwelten* of infrastructure: A stroll along (and inside) Phnom Penh’s sewage pipes. Jinbun (Nihon Jinbun Kagakkai) 47: 147–159.

[bibr20-25148486251371535] Joyce P (2003) The Rule of Freedom: Liberalism and the Modern City. London: Verso.

[bibr21-25148486251371535] Khmaladze I (1985) Landshapt’uri Khelovneba. Tbilisi: Khelovneba.

[bibr22-25148486251371535] K-me . (1903) Kutaisi. Iveria 1903(240): 2–3.

[bibr23-25148486251371535] Larkin B (2013) The politics and poetics of infrastructure. Annual Review Of Anthropology 42(1): 327–343.

[bibr24-25148486251371535] Latour B (1993) We Have Never Been Modern. Cambridge, MA: Harvard.

[bibr25-25148486251371535] Manning P (2012a) Semiotics of Drinks and Drinking. London: Continuum Press.

[bibr26-25148486251371535] Manning P (2012) Strangers in a Strange Land. Boston: Academic Studies Press.

[bibr27-25148486251371535] Manning P (2022) Flânerie in text and city: The heterogeneous urban publics of the Georgian feuilleton. Journal of Linguistic Anthropology 32(3): 585–606.

[bibr28-25148486251371535] Manning P (2023) The “poetic chaos” of gardens and genres in colonial Tbilisi. The International Journal of Middle East Studies 55: 498–516.

[bibr29-25148486251371535] Meneley A (2019) Walk this way: Fitbit and other kinds of walking in Palestine. Cultural Anthropology 34(1): 130–154.

[bibr30-25148486251371535] Meneley A (2025) Walking and perceptions of danger in various cities. *City & Society *37(2): e70010.

[bibr31-25148486251371535] Mgherabadze P (1908) Kutaisis bulvari. Eshmak'is Matrakhi 32: 4.

[bibr32-25148486251371535] Moagarak’e . (1903) Ts’erili Telavidan. Tsnobis Purtseli 1903 (2199): 3.

[bibr33-25148486251371535] Morson GS (1981) The Boundaries of Genre. Evanston: Northwestern University Press.

[bibr34-25148486251371535] Naraghi AR (2023) A Social History of Modern Tehran. Cambridge: Cambridge University Press.

[bibr35-25148486251371535] Nihil . (1894) Kutaisis “Gulvardi”. K’vali 1894(34), 4–7.

[bibr36-25148486251371535] Olmsted FL (2015 [1868]) Address to the Prospect Park scientific association. In: Writings on Landscape, Culture and Society. NYC, NY: Library of America, 429–439.

[bibr37-25148486251371535] Orbeliani S-S (1949) Kartuli Leksik’oni. Sakartvelos SSR: Tbilisi.

[bibr38-25148486251371535] Otter C (2007) Making liberal objects: British techno-social relations 1800–1900. Cultural Studies 21(4–5): 570–590.

[bibr39-25148486251371535] Patarkalashvili TK (2015) Urban and peri-urban forests of Tbilisi. Annals of Agrarian Science 13(1): 79–83.

[bibr40-25148486251371535] Patarkalashvili TK (2017) Urban forests and green spaces of Tbilisi and ecological problems of the city. Annals of Agrarian Science 15(2): 187–191.

[bibr41-25148486251371535] Pirtskhalava S (1988) Monogebata Purtslebi. Tbilisi: Sabch'ota Sakartvelo.

[bibr42-25148486251371535] Power E (2005) Human–nature relations in suburban gardens. Australian Geographer 36(1): 39–53.

[bibr43-25148486251371535] Sano . (1889) Pelet’oni: Kartvelta shoris. Iveria 1889(103): 1–2.

[bibr44-25148486251371535] Sasoplo Gazeti. 1912. Kutaisi . *Sasoplo Gazeti* *.* 1912(3): 4.

[bibr45-25148486251371535] Schmerling O (1903) Kutaisis gasartobi . Tsnobis Purtseli Suratiani Damat’eba 143: 4.

[bibr46-25148486251371535] Shling O. I. [Oscar Schmerling] (1916) *Int'erzoologiuri seirnoba Telavis bulvarshi. * *Eshmak'is Matrakhi* 31: 1.

[bibr46a-25148486251371535] Sigua A (1980) *Mitropane Laghidze* . Tbilisi.

[bibr47-25148486251371535] Skandarnova G (1879) Telavis mukhambazi. In Skandarnova G (ed) Msunagi-k’tsis Tskhovreba 67–69 A.A. Tbilisi: Mikhel’sona.

[bibr48-25148486251371535] Skandarnova G (1914) Allaverdi! Iakhsholdi!. Kutaisi: Karnakhovi.

[bibr49-25148486251371535] Skandeli N [Niko Nikoladze] (1871). Pelt’oni. Droeba 1871(19): 1.

[bibr50-25148486251371535] Sommer A-L (2007) Nature choreographed: The 18th Century garden as a knowledge-generating feature. https://webdoc.sub.gwdg.de/edoc/ia/eese/artic27/sommer/4_2007.html

[bibr51-25148486251371535] Tsereteli G (1887) Edisher Kelbakiani. Iveria. 1887(93): 1–2.

[bibr52-25148486251371535] Von Uexküll J (2010) The theory of meaning. In: Favareau D (eds) Essential Readings in Biosemiotics. Anthology and Commentary, London: Springer, 81–114.

[bibr53-25148486251371535] Walcher HA (1997) Between paradise and political capital: The semiotics of Safavid Isfahan. Middle Eastern Natural Environments Journal 103: 330–338.

[bibr54-25148486251371535] Warner M (2002) Publics and counterpublics. Public Culture 14(1): 49–90.

[bibr55-25148486251371535] Watelet CH (2003) Essay on Gardens: A Chapter in the French Picturesque. Translated by Samuel Danon. Philadelphia: University of Pennsylvania Press.

